# Bridging Gaps in Telemedicine Education in Romania to Support Future Health Care: Scoping Review

**DOI:** 10.2196/66458

**Published:** 2025-05-14

**Authors:** Mircea Adrian Focsa, Virgil Rotaru, Octavian Andronic, Marius Marginean, Sorin Florescu

**Affiliations:** 1 Medical Informatics and Biostatistics Department, Victor Babeș University of Medicine and Pharmacy Timișoara, Timisoara, Romania; 2 Victor Babeș University of Medicine and Pharmacy Timișoara, Eftimie Murgu 2 Sqr, Timișoara, 300041, Romania, 40 722343734; 3 General Surgery Department and Innovation and eHealth Center, Carol Davila University of Medicine and Pharmacy, Bucharest, Romania; 4 Public Health Authority, Brasov, Romania; 5 Orthopedics-traumatology II, Victor Babeș University of Medicine and Pharmacy Timișoara, Timisoara, Romania

**Keywords:** telemedicine, digital health, healthcare education, micro-credentials, scoping review, health education, Romania, future healthcare, telehealth, healthcare providers, technologies, digital literacy, healthcare system, quality care

## Abstract

**Background:**

Telemedicine is a key element of modern health care, providing remote medical consultations and bridging the gap between patients and health care providers. Despite legislative advancements and pilot programs, the integration of telemedicine education in Romania remains limited. Addressing these educational gaps is essential for preparing current and future medical professionals to effectively use telemedicine technologies.

**Objective:**

This study aimed to evaluate the current state of telemedicine education for medical professionals in Romania, focusing on the integration of diagnostic and therapeutic capabilities into medical curricula, identifying the challenges and opportunities, and providing recommendations for improving telemedicine education.

**Methods:**

A scoping review was conducted following Arksey and O’Malley’s framework. Peer-reviewed articles from 2019 to 2023 were identified using databases such as PubMed and Scopus. Additional gray literature was reviewed to provide a comprehensive understanding of telemedicine education in Romania. Data were thematically analyzed to extract key findings and recommendations.

**Results:**

The review identified significant progress in the legislative and infrastructural aspects of telemedicine in Romania, but highlighted gaps in integrating telemedicine education into curricula for medical professionals and other health care practitioners directly involved in telemedicine practices. While some universities have included telemedicine components, dedicated telemedicine courses and hands-on training remain insufficient. Barriers include a lack of infrastructure, digital literacy, and practical exposure to telemedicine technologies.

**Conclusions:**

For telemedicine to be effectively integrated into Romania’s health care system, medical education must be adapted to include comprehensive telemedicine training. Recommendations include enhancing digital literacy, fostering public-private partnerships, and incorporating telemedicine into undergraduate and continuous professional education programs. These efforts are essential for improving healthcare access and quality through telemedicine.

## Introduction

Telemedicine in Romania has undergone significant development over the years, marked by early pilot projects, legislative advancements, and a growing recognition of its potential to transform health care delivery. Given the rapid advancements in digital health globally, understanding and improving telemedicine education in Romania will not only enhance national health care delivery but also offer insights for similar health care systems transitioning to more digital practices.

For the purposes of this study and in accordance with Romanian legislation, telemedicine is defined as the use of digital technologies to deliver medical acts such as diagnosis, treatment, and therapy performed exclusively by licensed medical professionals. This differs from telehealth, which encompasses broader health-related services, including health education, prevention, and administrative activities.

While telemedicine inherently involves medical acts performed by licensed clinicians, it relies on a collaborative team, including nonclinical professionals, to ensure efficient and comprehensive care delivery.

Several interdependent factors, including educational competencies, regulatory policies, technological infrastructure, and institutional readiness shape telemedicine education. This study adopts a theoretical framework that integrates competency-based medical education, digital health policies, and workforce development strategies to evaluate the current state of telemedicine training in Romania. The framework is informed by internationally recognized guidelines, such as the World Health Organization (WHO) Digital Health Competency Framework (DHCF), the International Society for Telemedicine & eHealth (ISfTeH) guidelines, and the European Health Telematics Association (EHTEL) recommendations, which outline essential skills and knowledge areas for telemedicine practitioners.

Within this framework, telemedicine competencies are influenced by both regulatory structures and digital readiness, which shape how educational programs can be effectively implemented. While Romania has made legislative progress in supporting telemedicine, educational curricula remain inconsistent, lacking standardized competencies and hands-on training opportunities. Furthermore, limited technological infrastructure and digital literacy among both professionals and patients present additional challenges. By assessing these dimensions, this study identifies the current gaps in telemedicine education and proposes targeted recommendations to improve training programs, ensuring alignment with international best practices.

The journey began in 2001 when the Romanian Space Agency (ROSA) launched the Demonstrative Pilot of Telemedicine. This project, a significant milestone in the country’s health care history, focused on diagnostic, clinical, and educational applications, serving as a pioneering effort to explore the capabilities of telemedicine in the country. In 2003, further progress was made in establishing the Romanian Association for Telemedicine and Space Applications for Health (ATASS), which aimed to promote and develop telemedicine technologies [1].

A significant legislative milestone came in 2018 when telemedicine was formally incorporated into Romanian law through Government Emergency Ordinance number 8/2018, which amended Health Reform Law 95/2006. This legislative change aimed to address the chronic shortage of medical personnel, particularly in remote areas, and ensure more equitable access to healthcare services.

The COVID-19 pandemic in 2020 acted as a catalyst for telemedicine’s rapid adoption. In response to the crisis, the Romanian government took swift and decisive action to establish a regulatory framework for telemedicine services. Government Decision 252/2020 and subsequent ordinances laid the groundwork for telemedicine during states of emergency and beyond. These regulations facilitated various telemedicine services, including teleconsultation, tele-expertise, teleradiology, and telemonitoring. By 2022, the regulatory framework had further evolved with Government Decision 1133/2022 [2], which approved comprehensive implementation norms for telemedicine. This decision standardized procedures for scheduling remote appointments, protecting data privacy, and setting up payment mechanisms through the National Health care Insurance House. These measures ensured that telemedicine services could be provided seamlessly and securely, enhancing their integration into the healthcare system.

In the last 2 years, significant financial investments have supported the expansion of telemedicine in Romania. The recovery and resilience plan allocated substantial funds, including approximately €100 million for telemedicine support and €300-€400 million for hospital digitalization. These investments underscored the government’s commitment to advancing telemedicine as a critical component of health care delivery.

Despite these significant financial investments, the low levels of health and digital literacy among Romanian citizens present substantial barriers to the successful adoption and utilization of these technologies. Digital literacy for the public refers to the ability to access, understand, and use digital technologies for obtaining health information and services. For health care professionals, digital literacy extends to proficiency in using digital tools for clinical care, such as eHealth records, telemedicine platforms, and data privacy protocols.

Eurostat statistics [3] indicate that only 40% of Romanians use the internet to search for health information, significantly below the European Union average of 55%. This discrepancy highlights the role of education in digital engagement, with just 17% of individuals with low educational attainment using the internet for health purposes, compared with 41% of those with medium education and 66.5% of highly educated individuals.

The first cross-sectional study on health literacy in Romania [4] underscores the challenges faced by the population in processing health information. Approximately 21.6% of respondents found it difficult to protect themselves from illness based on health information provided by the media. Moreover, 7.5% of participants demonstrated inadequate health literacy, and 33.2% had problematic health literacy, leaving a majority (59.2%) with sufficient health literacy. Key determinants of health literacy included age, gender, education, and self-reported health status, while the residential area did not appear to influence health literacy levels. These findings underscore the considerable gaps in both health and digital literacy among the Romanian population.

Moreover, significant gaps still need to be addressed in integrating telemedicine education effectively within medical curricula, particularly in ensuring that current and future medical professionals are adequately prepared to leverage these technologies in practice. Most medical universities and medical schools have started incorporating telemedicine into their curricula, mainly as part of the medical informatics discipline, aiming to familiarize future health care professionals with digital health tools and focusing on both telemedicine’s technical and ethical aspects. Professional development opportunities have also expanded, with continuous education programs incorporating modules on telemedicine. Online training platforms and workshops have become vital resources for health care providers, helping them stay updated with the latest advancements and best practices in telemedicine. Beyond serving as a means of continuing education, these platforms often provide essential initial training for health care professionals new to telehealth, equipping them with foundational knowledge and skills.

Telehealth competency, encompassing knowledge, skills, and attitudes essential for effectively delivering care via telemedicine, is increasingly recognized as a critical aspect of modern health care practice. However, in Romania, health care professionals often lack structured and standardized training in telemedicine, resulting in gaps in areas such as conducting teleconsultations, ensuring data security, and communicating effectively with patients in virtual settings. These gaps highlight the need for targeted educational interventions to prepare health care professionals for the demands of telemedicine.

The scope of this study is confined to telemedicine, which involves clinical activities performed by physicians and other licensed medical professionals, ensuring a clear distinction from the broader concept of telehealth.

The aim of this scoping review is to evaluate the current state of telemedicine education in Romania, identify the challenges and opportunities associated with its integration into medical curricula, and provide recommendations for improving telemedicine education. Specifically, this study maps existing telemedicine education initiatives, assesses barriers to implementation, and proposes strategies to enhance training programs for health care professionals.

## Methods

### Study Design

This study used a scoping review methodology to comprehensively explore the current landscape of telemedicine education in Romania. The study specifically evaluates educational approaches for medical professionals performing telemedicine, addressing clinical activities such as diagnosis, therapy, and patient management. Telemedicine education evaluated in this study encompasses competencies applicable to multiple specialties, including primary care, chronic disease management, and specialized services such as telemonitoring and telerehabilitation. A scoping review was chosen for its ability to map key concepts, types of evidence, and gaps in research related to a defined area or field of interest, particularly in complex or under-reviewed topics. This approach is especially suitable for telemedicine education in Romania, given its rapidly evolving nature and the need to synthesize diverse sources of information

### Research Question

The primary research question guiding this scoping review was: “What is the current state of telemedicine education in Romania, and what are the key challenges and opportunities for its integration into medical curricula?”. This question was formulated to encompass the broad scope of telemedicine education, including formal educational programs, professional development initiatives, and digital literacy efforts.

### Literature Search Strategy

A systematic search of peer-reviewed journal papers was conducted across multiple databases, including PubMed, Scopus, Web of Science, and Google Scholar. The search covered articles published between January 2019 and December 2023. The following keywords and their combinations were used: telemedicine, telehealth, digital health, medical education, telemedicine education, Romania, eHealth, and digital literacy.

Additional filters were applied to include only papers available in English or Romanian, with a focus on education, telemedicine implementation, and health care policy in Romania. Papers were limited to those that addressed telemedicine within the context of health care education, the use of digital tools in clinical training, and barriers or facilitators to telemedicine adoption.

In addition to peer-reviewed papers, relevant gray literature was included in the scoping review. Sources of gray literature encompassed reports from governmental and nongovernmental organizations, policy briefs, and institutional documents related to telemedicine education in Romania. These documents were identified through searches of online repositories and institutional websites, and they provided critical context on legislative developments, pilot projects, and educational initiatives that may not have been extensively covered in academic databases.

### Study Selection

The study selection process involved 2 independent researchers (MF and VR) who screened titles, abstracts, and full texts to ensure alignment with the inclusion and exclusion criteria. Any disagreements were resolved through discussion, and when consensus could not be reached, a third researcher reviewed the articles to make the final decision. The process was facilitated using Rayyan platform, allowing efficient tracking and documentation of the selection process.

The initial search yielded 105 journal papers, screened for relevance based on titles and abstracts. Articles that focused on telemedicine in clinical practice without addressing educational aspects were excluded. After this preliminary screening, 35 papers remained for full-text review. A further screening based on inclusion and exclusion criteria led to the identification of 19 papers deemed highly relevant to the study’s aims.

### Eligibility Criteria

The inclusion and exclusion criteria are listed in Textbox 1.

Textbox 1. **Inclusion and exclusion criteria**

**Inclusion criteria**

- Studies published between 2019 and 2023.
- Studies addressing telemedicine education or digital literacy training in medical or health care fields.
- Studies conducted in or relevant to the Romanian context.

**Exclusion criteria**

- Articles focusing exclusively on telemedicine in clinical practice without reference to education.
- Studies not available in English or Romanian.

### Data Extraction

For each of the 19 selected peer-reviewed papers, data were extracted using a standardized data charting form, which included the following variables: (1) author(s), publication year, and country of origin; (2) study design (eg, qualitative, quantitative, and mixed methods); (3) the focus of the study (eg, telemedicine education, barriers to digital health adoption, and telemedicine curriculum development); (4) key findings relevant to telemedicine education, digital literacy, and health care training; and (5) recommendations for telemedicine integration into medical education.

### Data Analysis

Two independent researchers conducted a thematic analysis to identify recurring patterns and themes within the selected literature. An inductive approach allowed themes to emerge naturally from the data. The analysis involved coding and categorizing data using manual methods, followed by a review of the themes to ensure consistency and relevance. The themes were grouped into broad categories, such as the current status of telemedicine education in Romania, barriers to telemedicine education, opportunities for development, and digital literacy challenges.

### Ethical Considerations

Since this study was based on a review of publicly available literature, no ethical approval was required. However, all articles were reviewed with a commitment to academic integrity and transparency, and any potential conflicts of interest were disclosed.

## Results

### Included Studies

**Figure 1.**
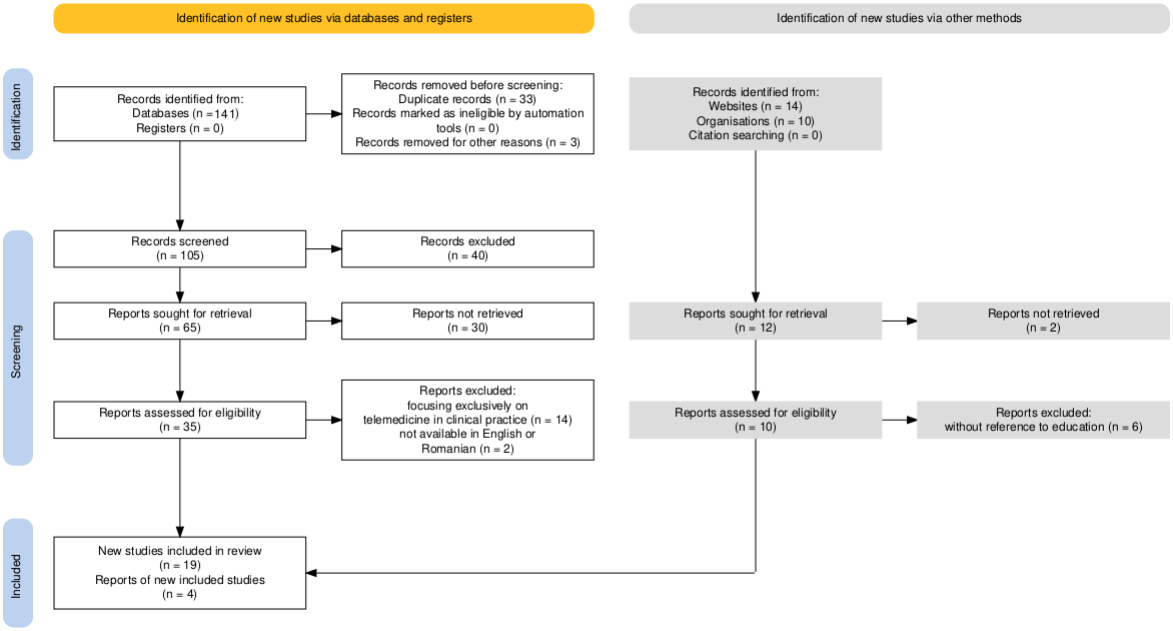
PRISMA (Preferred Reporting Items for Systematic Reviews and Meta-Analyses) flowchart for identification and selection of studies.

The initial search yielded 105 journal papers, screened for relevance based on titles and abstracts. Articles that focused on telemedicine in clinical practice without addressing educational aspects were excluded. After this preliminary screening, 35 papers remained for full-text review. A further screening based on inclusion and exclusion criteria led to the identification of 19 papers deemed highly relevant to the study’s aims (Figure 1 and Checklist 1) .

The results presented are based on themes identified through inductive thematic analysis, which highlighted key areas such as the integration of telemedicine into curricula, barriers to adoption, and digital literacy as a critical enabler for telemedicine education.

### Current State of Telemedicine Education in Romania

The data collected and synthesized in Table 1 offers a comprehensive overview of recent research in telemedicine, eHealth, and artificial intelligence (AI)-based health care interventions in Romania. It includes various study designs such as cross-sectional, prospective, case-control, and system architecture studies and spans diverse health care topics, including telemedicine-driven rehabilitation, the impact of virtual communities on telemedicine adoption, and the integration of AI and Internet of Things (IoT) technologies in health care. The analysis covers key aspects of telemedicine’s role during and post–COVID-19 and its relevance for both chronic diseases and mental health management. The studies underscore the positive reception of telemedicine in various medical fields, from diabetes management to cardiac rehabilitation, with the technology being especially vital during the COVID-19 pandemic. However, challenges such as digital literacy, accessibility, and infrastructure remain.

While Table 1 provides a summary of the reviewed studies, the specific components of telemedicine education and digital literacy training are discussed in greater detail within the following subsection, to align with the thematic approach of this scoping review.

**Table 1.** Overview of recent research in telemedicine, eHealth, and artificial intelligence–based health care interventions in Romania [5-23].

PMID	The focus	Key findings	Recommendations
35879930 [5]	Openness of medical students to telemedicine	Moderate-to-high acceptance, need for practical exposure	Integrate telemedicine into medical curricula, enhance digital literacy
35935629 [6]	Evaluating telemedicine benefits for cardiovascular patients during COVID-19.	Telemedicine facilitated patient management, including medication adjustments, but barriers like data security and reimbursement need addressing.	Improve telemedicine frameworks and regulations for cardiovascular care beyond the pandemic.
37297692 [7]	Perceptions of telemedicine among health care professionals	Positive outlook, concerns over digital literacy	Increase digital education for health care professionals
37510969 [8]	Telemedicine-driven pulmonary rehabilitation for post–COVID-19	Significant improvement in physical and mental health	Integrate telerehabilitation into postacute COVID-19 management
36141685 [9]	AI^a^ in eHealth, telemedicine, and remote monitoring	AI advances in eHealth and telemedicine	Enhance integration of AI into eHealth for better health care services
36141899 [10]	Home-based robotic cardiac telerehabilitation system	RoboTeleRehab system is feasible, positive feedback	Further testing on cardiac patients, integration into rehabilitation programs
33923842 [11]	Blockchain-enabled framework for mHealth^b^ systems	Improves security, transparency, and immutability	Implement blockchain for secure patient data management in mHealth
35062129 [12]	AI in primary care and telemedicine	AI aids in primary care diagnosis, treatment, and decision-making	Enhance AI systems to support primary care and telemedicine workflows
36141297 [13]	Impact of virtual communities on telemedicine usage	Virtual communities influence patient satisfaction and usage intention	Use virtual communities to promote telemedicine services
35954526 [14]	Adoption of eHealth and mHealth for mental health	Accessibility and data security are critical for adoption	Improve accessibility and data security in digital mental health tools
36556027 [15]	Management of dilated cardiomyopathy during COVID-19 using telemedicine	Telemedicine maintains clinical stability in patients with home monitoring	Use multiparametric home monitoring to manage dilated cardiomyopathy during crises
33953605 [16]	Perception of Romanian family doctors on telemedicine during COVID-19	Positive view, but tele-diagnostic challenges and time constraints	Training for family doctors and continued telemedicine reimbursement
34898981 [17]	Assessing patient adherence to telemedicine in diabetes	Developed reliable and valid tool for telemedicine adherence in diabetes care	Use the instrument to optimize telemedicine platforms based on patient needs
35150518 [18]	Attitudes toward eHealth during the COVID-19 pandemic	Negative attitudes in Greece due to forced usage	Address technical skills gaps and improve ease of access
37372846 [19]	Telemonitoring for cardiovascular disease during and post-COVID-19	Telemedicine improved cardiovascular prevention during the pandemic	Universal access to home telemonitoring for high-risk cardiovascular patients
36900948 [20]	Virtual assistant in cardiac rehabilitation	Similar results for virtual versus in-person rehabilitation	Optimize virtual assistants for cardiac rehab
33800728 [21]	IoT^c^-based biometric monitoring system for elders	Scalable solution for monitoring and cognitive assessment	Expand to predictive analysis for cognitive and physiological data
35979169 [22]	Telerehabilitation for Parkinson disease	Improved walking performance using telerehabilitation	Expand telerehabilitation for other neurorehabilitation settings
31407668 [23]	Use of telemedicine for rare diseases in Romania	Telemedicine improves access to rare disease care	Increase telemedicine use for remote consultations in rare diseases

a AI: artificial intelligence.

b mHealth: mobile health.

c IoT: Internet of Things.

### University Programs and Curricula

In Romania, prominent medical universities in Timisoara, Cluj-Napoca, Iasi, and Bucharest have incorporated telemedicine and digital health into their educational programs. These changes were particularly accelerated following the COVID-19 pandemic, which highlighted the need for digital health competencies among health care professionals. New curricula encompass various aspects, such as telemedicine applications, electronic health records (EHRs), and data management strategies. Despite these inclusions, the development of dedicated disciplines and laboratory classes specific to telemedicine remains ongoing.

While telemedicine education in Romania has introduced theoretical foundations and basic digital health tools, it remains limited in scope and practical application. Key gaps include the lack of hands-on training, comprehensive and stand-alone telemedicine courses, and interprofessional education initiatives. Furthermore, advanced topics such as AI and patient engagement strategies remain underexplored, emphasiing the need for structured and innovative approaches to telemedicine education.

On October 14, 2023, the Center for Innovation and e-Health at the University of Medicine and Pharmacy “Carol Davila” in Bucharest hosted a specialized course titled “Telemedicine - Current Information and Skills.” [24]. This initiative covers fundamental theoretical concepts of telemedicine, including applicability and legal frameworks, alongside practical skills for using associated technologies.

Many health care professionals participate in continuing medical education (CME) programs to stay updated on telemedicine. These programs focus on skills for telemedicine platforms, data privacy laws, and improving remote patient interaction. The Digital Innovation Zone Association [25], affiliated with the North-East Regional Development Agency, offers comprehensive 1- to 2-month programs delivered in a hybrid online and on-site format.

A significant milestone in telemedicine education within the country was marked by the launch of the Erasmus+ project “TEAM: Supporting Innovation in Telemedicine Education with Cross-European Collaboration” in November 2023. The University of Medicine and Pharmacy “Victor Babes” in Timisoara is a principal participant in this consortium, which also includes partners from Belgium, Slovenia, Croatia, Greece, and Ukraine. The project aims to develop adaptable learning pathways that conform to international best practices, focusing on surmounting challenges such as limited digital literacy and fostering cross-sectoral cooperation. Targeting higher education students in health care and IT, educators, and institutions, the project also extends its reach to health care and IT professionals and policymakers. One of the primary outcomes of this initiative is the establishment of flexible microcredentials in Telemedicine designed to enhance proficiency in digital health among students and professionals alike.

### Professional Development Workshops and Seminars

Universities and health care institutions in Romania proactively conduct workshops and seminars to offer practical training in telemedicine technologies. These educational sessions frequently use case studies and practical simulations to enrich the learning experience. They cover a spectrum of topics, from fundamental telemedicine principles to advanced applications such as the integration of AI tools in clinical settings.

Across Romania, hospitals and medical associations also organize workshops and seminars aimed at practicing health care professionals. These sessions foster proficiency in telemedicine platforms, digital communication skills, and data security. Participants, including doctors, nurses, and ancillary staff, benefit from the self-paced learning environment these workshops provide. They typically include hands-on sessions where attendees can interact with telemedicine software, acquire best practices for conducting virtual consultations, and gain insights into the legal and ethical dimensions of remote health care.

A notable initiative was the establishment of the ROHEALTH cluster in 2015, which brought together various entities within the health and bioeconomy sectors to enhance their competitive edge. This cluster supports an online platform offering diverse courses and webinars, including specialized offerings such as “eTELEDOC, emergency telemedicine” [26]. These resources aim to bolster the competencies of health care professionals in the evolving landscape of telemedicine.

### Digital Literacy Programs

Several nongovernmental organizations offer training programs and workshops to enhance digital literacy among health care providers. Initiated in 2022 by PALMED, the Patronage of Private Medical Service Providers, the project titled “Digital Skills for Employees - Support for SMEs in the Health Sector to Assimilate Technologies and Develop Telemedicine Services (TELMed)” is a notable endeavor under the Human Capital Operational Program for 2014–2020 [27]. This project specifically aims to augment the digital competencies of personnel across 35 small and medium-sized enterprises in the health care sector, including hospitals, clinics, offices, and laboratories. By enhancing these skills, the initiative not only supports the adaptation and expansion of telemedicine activities but also prepares the workforce for advancements related to Industry 4.0 and smart specialization areas.

### Challenges in Telemedicine Education

The challenges identified in this section were derived from thematic analysis of the 19 peer-reviewed papers and relevant gray literature, including institutional reports and policy briefs. These sources highlighted common barriers such as limited digital literacy among health care professionals, the lack of standardized curricula, and inadequate infrastructure for telemedicine training.

The pandemic propelled telemedicine into prominence, shedding light on Romanian family doctors’ diverse experiences and perceptions [16]. Over a quarter of general practitioners reported that remotely addressing patients’ health care needs was more manageable, demonstrating adaptability to telemedicine modalities. Nevertheless, challenges such as the time-intensive nature of teleconsultations, diagnostic uncertainties, and patients’ difficulties with technology have surfaced. These issues highlight the critical need for specialized training programs in telemedicine for both health care professionals and patients to mitigate disruptions in health care delivery effectively.

Furthermore, the moderate-to-high acceptance of telemedicine among Romanian medical students emphasizes the necessity of integrating telemedicine education early in their medical training. Telemedicine fundamentally transforms the patient-physician relationship, requiring physicians to develop new communication skills, known as “webside manner.” The methodological norms for telemedicine services in Romania also emphasize the importance of well-trained professionals who can navigate legal, ethical, business, and practical challenges. Training should start at the undergraduate level and continue through all professional stages for medical staff in all specialties.

While the primary objective of this paper is to explore telemedicine education for health care professionals, patient experiences with telemedicine offer indirect yet critical insights. These experiences highlight areas where health care providers may require additional training, such as building virtual rapport, managing technological issues, and addressing patient concerns about telemedicine efficacy and privacy. Integrating these considerations into training programs can better align telemedicine education with real-world practice.

In 2021, a study [17] evaluating the desirability, acceptability, and adherence to telemedicine among diabetes patients underscores the need for educational programs. Such initiatives targeting patients are essential to foster a positive perception and readiness to use telemedicine services, particularly in chronic conditions like diabetes, where continuous care and monitoring are essential. Patients, particularly those managing chronic diseases, require thorough education to use virtual technology effectively, including understanding how to operate software and hardware, such as mobile communication devices and other digital interfaces essential for remote health care.

## Discussion

### Principal Findings

This scoping review identified significant gaps in telemedicine education in Romania, despite recent legislative and infrastructural advancements supporting telemedicine adoption. While some medical universities have incorporated telemedicine content into their curricula, there remains a lack of structured, hands-on training and dedicated telemedicine courses. The study also highlighted key barriers, including limited digital literacy among health care professionals, insufficient policy support for mandatory telemedicine training, and a lack of standardized competency frameworks. These findings underscore the need for formalized telemedicine education initiatives, integrated into both undergraduate and CME programs, to enhance digital health preparedness among health care providers.

The findings of this scoping review highlight the significant progress and persistent challenges in telemedicine education in Romania. This discussion will interpret these results, explore their implications, and propose strategies for advancing telemedicine education in the country. To advance telemedicine in Romania, several strategic directions need to be pursued.

Medical schools must develop comprehensive programs that include hands-on training with telemedicine platforms and technologies, such as conducting teleconsultations or using telemonitoring devices in simulated or real clinical environments. The launch of specialised courses and international collaborations, such as the Erasmus+ TEAM project, demonstrates a commitment to enhancing telemedicine education.

In Romania, health care professionals are required by law to participate in CME programs to retain their licenses. While telemedicine-specific education is not yet a mandatory component of these requirements, its growing integration into CME programs highlights the recognition of telemedicine as an essential skill set for modern medical practice. This approach aligns with recommendations from the EHTEL, which emphasizes the importance of ongoing training in digital health for all health care providers [28]. The involvement of industry clusters like ROHEALTH in providing specialized webinars indicates a promising collaboration between academia and industry.

However, the review also suggests that these efforts may not be sufficient to meet the rapidly evolving needs of the health care system. There appears to be a need for more structured, comprehensive, and widely accessible professional development programs in telemedicine. While beneficial for updating specific skills, self-directed CME is often insufficient in providing a holistic understanding of telehealth practice. Without structured education, health care professionals may lack critical knowledge of professional telehealth standards, guidelines, and best practices, placing them at risk of learning by trial and error. Comprehensive, formalized training programs are essential to equip professionals with the competencies needed to deliver high-quality, safe, and effective telehealth care.

The identified gaps in digital literacy among both health care providers and patients represent a significant barrier to the effective implementation of telemedicine. The initiatives aimed at enhancing digital skills, such as PALMED’s “Digital Skills for Employees” project, are steps in the right direction. These efforts align with European Union–wide initiatives like the Digital Education Action Plan (2021‐2027) [29], which emphasizes the importance of digital skills across all sectors, including health care. However, these efforts need to be scaled up and integrated more systematically into both medical education and public health initiatives. The review also highlights the need for patient education in telemedicine, particularly for managing chronic conditions. In addition, robust policy support, increased public awareness, and education are crucial for the effective implementation of telemedicine, ultimately improving health care access and outcomes across Romania.

To fully realize the benefits of telemedicine, regulatory bodies in Romania should consider introducing mandatory education requirements for telemedicine practice. While technological proficiency is an important component of telemedicine education, health care professionals must also develop a broader range of competencies to provide effective telehealth care. These include clinical decision-making, patient communication, and understanding the ethical and legal dimensions of telemedicine. Incorporating recognized telemedicine competency frameworks into educational programs can ensure comprehensive preparation for future telehealth practitioners.

Telemedicine is pivotal in advancing integrated care by fostering coordination among health care professionals and enabling patient-centered approaches. To fully realize its potential, telemedicine education must include interprofessional education and training for collaborative care, equipping health care teams with the skills to work cohesively in delivering seamless and effective telehealth services [30].

The results highlight a diverse range of technologies relevant to telemedicine education, including mobile health platforms, IoT devices, EHR systems, and continuous monitoring technologies. Although only a minority of reviewed studies explicitly addressed AI, its inclusion underscores the importance of preparing professionals for future technological advancements. While AI is not yet pervasive in all areas of health care, equipping professionals with foundational knowledge will prepare them for its growing integration into clinical practice.

Creating a thorough telemedicine education program necessitates teamwork between health care professionals, educators, technology specialists, and policymakers. Collaborating can help ensure the curriculum meets health care system needs, includes cutting-edge telemedicine technologies, and prepares providers and patients for future health care delivery.

### Implications of Findings

To close the skills gaps, it is essential to develop educational programs that address the points mentioned below.

#### Improve Digital Literacy

Training must concentrate on enhancing the digital skills of health care professionals and patients so they can successfully navigate eHealth and mobile health platforms. While improving, digital literacy among Romanian health care professionals remains largely dependent on voluntary initiatives such as webinars, workshops, and seminars. This fragmented approach highlights the need for structured and comprehensive education programs to ensure that professionals are fully equipped to leverage telemedicine technologies effectively.

#### Integrates AI Technologies

Educational initiatives must cover foundational AI and machine learning (ML) concepts relevant to health care applications, including data privacy, ethical considerations, and interpreting AI-generated insights.

#### Develop New Skills

Training for telemedicine should include soft skills such as remote patient interaction, digital communication etiquette, and managing online patient relationships to adapt to remote health care dynamics. It also includes educating patients on how to use telemedicine services, getting ready for virtual visits, and handling their health data online.

#### Promote Trust in the Efficacy of Telemedicine

The research suggests the importance of increasing trust among health care providers and patients regarding telemedicine’s effectiveness. For that purpose, telemedicine training should cover not only the technical aspects but also the clinical relevance and influence on patient outcomes, especially in managing chronic conditions.

#### Deal With Ethical and Privacy Concerns

Telemedicine training should incorporate adherence to recognized telehealth and telemedicine standards and guidelines, such as GDPR (General Data Protection regulations) and those created by the International Society for Telemedicine and eHealth [31]. Training should address ethical issues, privacy concerns, legal regulations, patient privacy protection strategies, and ethical guidelines for virtual patient interactions, ensuring alignment with global best practices.

### Limitations and Future Directions

This scoping review, while comprehensive, has certain limitations. The focus on English and Romanian language publications may have excluded relevant studies in other European languages. In addition, the rapid evolution of telemedicine, particularly in response to the COVID-19 pandemic, means that some recent developments may not be fully captured in the published literature.

Future research should focus on (1) longitudinal studies assessing the long-term impact of telemedicine education on health care delivery and outcomes in Romania; (2) comparative analyses of telemedicine education approaches across different European countries, particularly comparing Eastern and Western European contexts; and (3) in-depth qualitative studies exploring the experiences and perspectives of medical students, health care providers, and patients regarding telemedicine education and implementation in the Romanian and broader international context.

### Conclusion

Despite significant advancements, telemedicine in Romania still faces challenges. Infrastructure deficiencies, digital literacy gaps, and regulatory hurdles remain significant obstacles. However, ongoing investments in infrastructure, education, and regulatory frameworks are expected to address these issues, paving the way for broader adoption of telemedicine and improved health care access across the country.

The integration of telemedicine into medical education in Romania is crucial for the future of health care delivery. By addressing the current challenges and learning from successful global models, Romania can enhance its telemedicine capabilities and ensure that health care providers are well-prepared to leverage telemedicine technologies to improve health care access and quality. Moving forward, efforts should focus on enhancing digital health literacy, optimizing telemedicine systems, and expanding the use of AI and IoT for more integrated health care.

The future of telemedicine in Romania looks promising. As the country continues to invest in telemedicine education and infrastructure, health care providers will be better prepared to leverage digital health technologies, ultimately enhancing the quality and accessibility of health care services for all Romanians.

## Supplementary material

10.2196/66458 Checklist 1 PRISMA-ScR checklist.

## Abbreviations

AI: artificial intelligence
ATASS: Association for Telemedicine and Space Applications for Health
CME: Continuing Medical Education
DHCF: Digital Health Competency Framework
EHR: electronic health record
EHTEL: European Health Telematics Association
GDPR: General Data Protection regulations
IoT: Internet of Things
ISfTeH: International Society for Telemedicine & eHealth
ROSA: Romanian Space Agency
WHO: World Health Organization
